# Osteochondroma of the pubic symphysis causing hematuria: a case report and literature review

**DOI:** 10.1186/s12894-020-00770-8

**Published:** 2021-01-06

**Authors:** Li-cheng Song, Qian Xu, Hui Li, Zhi-jun Li, Ya Li, Ya-fei Qin, Bao-long Wang, Hua-feng Zhang

**Affiliations:** 1 Department of Orthopaedic, Tianjin Medical University General Hospital, Anshan Road No. 154, Heping District, Tianjin, 300052 China; 2 School of Integrative Medicine, Tianjin University of Traditional Chinese Medicine, Tianjin, 301617 China; 3 Department of the Obstetrics and Gynecology, Tianjin Medical University General Hospital, Tianjin, 300052 China; 4 Department of Urology, Tianjin Medical University General Hospital, Anshan Road No. 154, Heping District, Tianjin, 300052 China

**Keywords:** Osteochondroma, Pubic symphysis, Hematuria, Case report

## Abstract

**Background:**

Osteochondroma is the most common benign bone neoplasm and is sometimes referred to as osteocartilaginous exostosis. The symptoms caused by osteochondroma are rare, especially the urogenital complications. Therefore, this tumour is sometimes misdiagnosed.

**Case presentation:**

This report described a 70-year-old woman with hematuria who was initially misdiagnosed with a bladder tumour in the outpatient department by a urologist. However, during cystoscopy, we found that the mass did not resemble a bladder tumor. Multidisciplinary approach with careful analysis of the imaging data suggested the diagnosis of osteochondroma. Open surgical excision of the mass was done and histology confirmed the diagnosis of benign osteochondroma. After 6 months of follow-up, the patient was still asymptomatic.

**Conclusions:**

This case illustrates that hematuria is caused by not only urogenital disease but also osteochondroma. We present this case to draw the attention of clinicians to osteochondroma of the pubic symphysis.

## Background

Osteochondroma, also called osteocartilaginous exostosis, is the most common benign bone neoplasm, accounting for 10–15% of all bone tumours and 45% of all benign bone tumours [1, 2]. However, it rarely causes complications unless the tumour is large or it is located in a crucial anatomical place [1]. Osteochondroma can present as solitary exostosis or as a part of hereditary multiple exostoses [2]. Clinical data indicate that urogenital symptoms (compression, pain, dyspareunia, haematuria, and urination difficulties) are rare. Here we report a case of osteochondroma of the pubic symphysis causing hematuria. This report intends to reinforce awareness of the disease among clinicians and to establish a clear diagnosis in the future.

## Case presentation

**Fig. 1:**
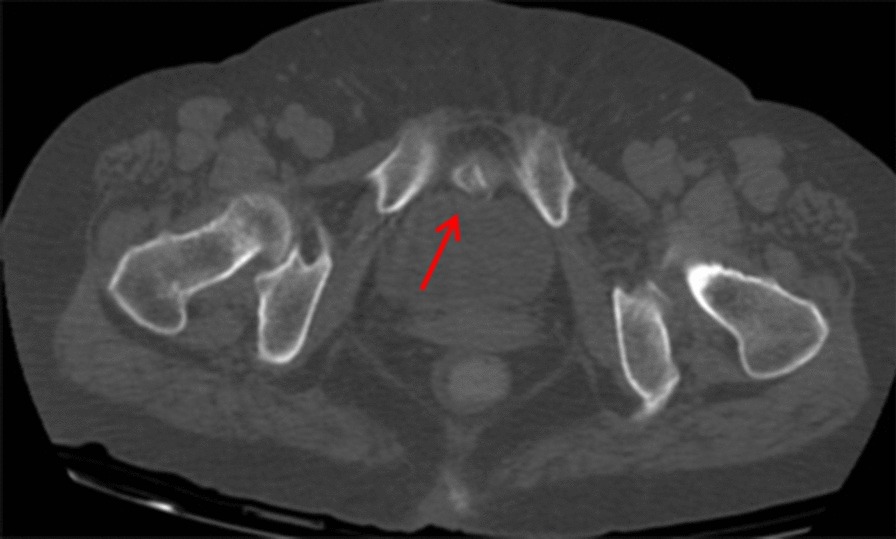
Primary abdominal CT showed a high-density mass in front of the bladder that compressed the antetheca (arrow)

A 70-year-old woman underwent routine physical examination 2 years ago, and the routine urine tests showed microscopic hematuria. Because of the absence of symptoms of gross hematuria and urgency, the patient did not take any further action or therapy. 3 months ago, routine urine tests still showed microscopic hematuria. Then, she went to the outpatient department for medical help from a urologist. The doctor first advised her to undergo a urinary ultrasound, and the result showed a low echogenic masses. Then, she underwent a abdominal computed tomography (CT), showed that there was a high-density mass anterior of the bladder that compressed the antetheca (Fig. 1). Combining the patient’s age, imaging findings and routine urine tests, the urologist made a primary diagnosis of a bladder tumour.

On admission, the patient’s physical examination was normal, without any significant pathological findings. The patient had a past medical history of hypertension. She denied smoking and drinking. Her family history was unremarkable. Routine urine showed that occult blood in the urine was 2+, the red blood cell count was 20/HPF (high-power field), the white blood cell count was 109/HPF, and the epithelial cell count was 6/HPF. Bacterial culture of the urine demonstrated no evidence of colony formation. Other laboratory data, such as routine blood tests, blood biochemistry and immune indexes, were normal.

**Fig. 2:**
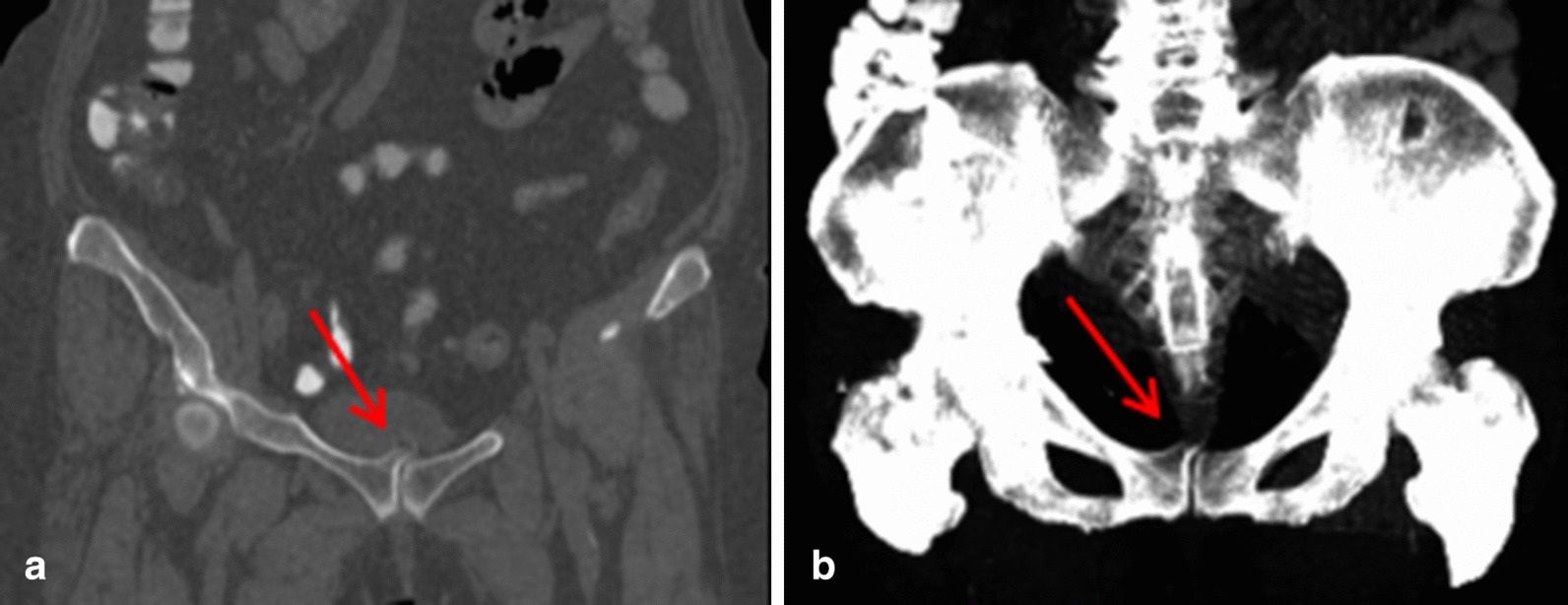
**a** Coronal CT showed that a lesion arose from the superior aspect of the right pubic symphysis and grew towards the upper left region, impinging on the bladder (arrow). **b** The 3D reconstruction of the pelvis showed that the marrow and cortex of the bony mass were continuous with those of the adjacent normal bone (arrow)

**Fig. 3:**
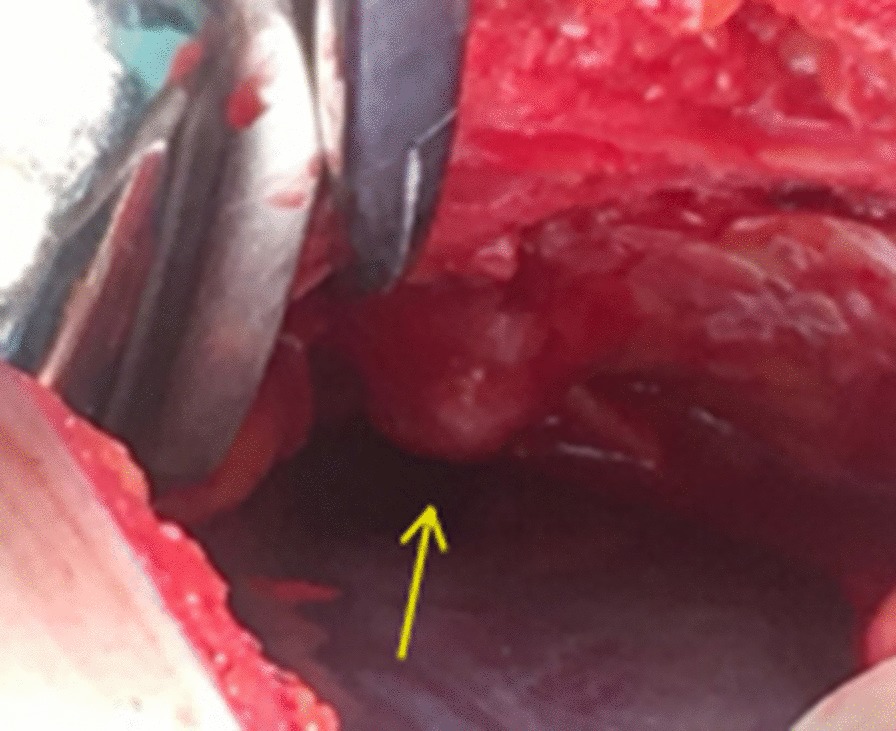
Intraoperative image showed an irregularly shaped bone mass approximately 1.5 × 1.0 × 0.6 cm in size in front of the bladder (arrow)

**Fig. 4:**
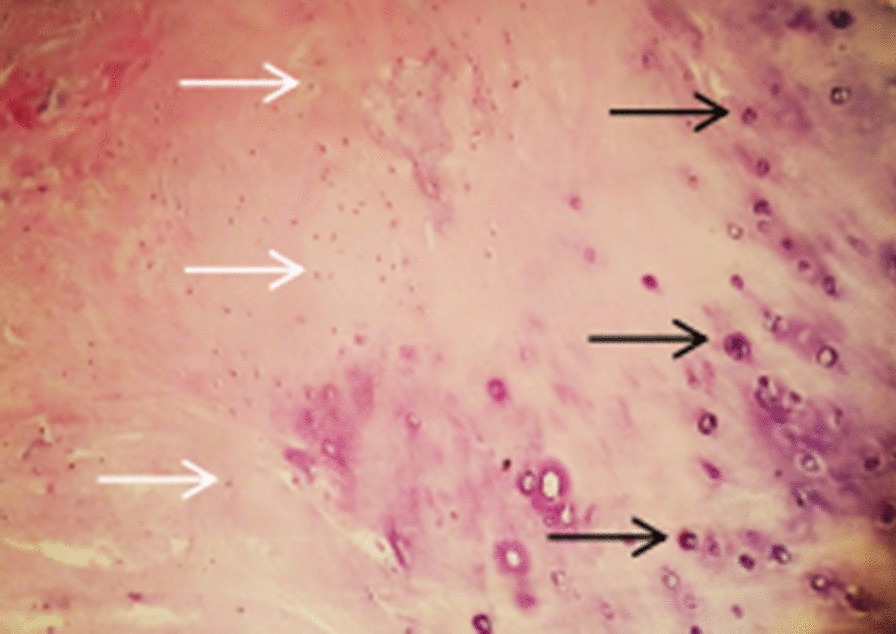
Haematoxylin and eosin staining showed that the chondrocytes in the cartilage lacuna (black arrow) and osteocytes (white arrow) were diffusely distributed (magnification, × 400)

Considering the patient's age and related high-risk factors, we arranged cystoscopy (OLYPUS) operation in the operating room. During the operation, we find that the lump was not inside but outside the bladder and that it compressed the bladder. For this situation, we immediately organized a multidisciplinary treatment (MDT). During the discussion, the orthopaedist proposed that coronary imaging and 3D reconstruction of the abdominal CT should be performed. Therefore, we performed coronary imaging and 3D reconstruction on the computer immediately (Fig. 2). According to the observation of the images, the orthopaedist made a diagnosis of osteochondroma. Our patient underwent surgery by the orthopaedist and urologist under general anaesthesia. During the operation, we found an irregular bone tumour approximately 1.5 × 1.0 × 0.6 cm in size on the pubic symphysis that compressed the anterior bladder wall (Fig. 3), We resected it completely. The postoperative biopsy of the mass indicated a benign osteochondroma (Fig. 4). After resection, the microscopic hematuria disappeared. After the 6-months follow-up, the symptoms were still absent.

## Discussion and conclusions

Osteochondroma is a benign tumour with uncertain aetiology. Osteochondroma is considered to be a developmental physeal abnormality rather than a primary bone neoplasm [3]. It is covered by a cartilaginous cap that ossifies with age to produce punctate or nodular calcification [4]. Plentiful calcification might appear on benign lesions. The marrow and cortex of osteochondroma are continuous with those of adjacent normal bone. We should be careful of the malignant transformation when the thickness of the cartilage cap exceeds 2 cm in adults and 3 cm in children [1]. The diameter of the lesion is often < 5 cm when diagnosed [5]. The growth of osteochondroma continues during the active growing period and stops in adolescence [6, 7]. Most of the symptomatic lesions are diagnosed between 10 and 20 years old due to increased skeletal growth, and the male:female ratio is approximately 2:1 [4–6].

A solitary osteochondroma usually forms at the metaphysis of a long tubular bone, particularly around the knee and at the lower femur and the upper tibia [5, 6]. Although lesions may be seen in the scapula, clavicle and ilium, their presence in the bones of the hand and foot as well as the pubic symphysis is rare [5]. Hereditary multiple exostoses is an autosomal-dominant disorder associated with the *EXT1* and *EXT2* genes [3, 5]. Only less than 1% of solitary osteochondromas transform to chondrosarcomas during later life, and the incidence of malignant changes rises to 10% in hereditary multiple exostoses (diaphyseal achalasia) [1, 4, 6, 8]. Thus, removal should be considered. It has been documented that the recurrence of osteochondroma is ~ 2% after resection [5].

The symptoms of patients usually come from mechanical irritation or compression of adjacent structures (soft tissues, bone, internal organs, peripheral nerves, spinal cord, and blood vessels), fracture, and malignant transformation [1, 9].

According to the literature, pelvic osteochondroma accounts for approximately 5% of all osteochondromas [10, 11]. Osteochondroma near the pubic symphysis is rare. We performed an extensive review of available reports about osteochondroma near the pubic symphysis from 1980 to the present (Table 1). Amis [7], Cardenas [12] and Bacha [13] described asymptomatic patients at the time of routine physical examination. Phillips [4] reported that exostosis arising from the superior aspect of the right pubic ramus had a close relationship with the prostate and bladder base. This is the only case report of haematuria caused by osteochondroma in the literature. Hoshimoto [6] described a 25-year-old woman with compression of the vagina and coital pain by the pubic bone. Amis [7] described a 23-year-old asymptomatic white man with a firm, enlarged and symmetrical prostate. However, this patient refused surgical intervention. Herode [3] described the reasons for resection as cosmetic, and the others opted for excision to relieve the relevant symptoms. Hence, appropriate radiographic and detailed preoperative evaluation must be carried out before any biopsy or surgical procedure to properly evaluate hard masses of the bony pelvis and to help develop a properly operative plan for management of any bone masses [5, 12].

**Table 1:** Available case reports of osteochondroma near the pubis symphysis

Author	Year	Age/gender	Site	Symptoms	Therapy	Follow-up	References
Kemal	2015	20/F	Pubic ramus	Groin pain, dysuria and obstructive urination	Resection	1 month	[2]
Herode	2015	18/F	Pubic ramus	Groin swelling	Resection	1 year	[3]
Phillips	1987	41/M	Pubic ramus	Hematuria	Resection	Not mentioned	[4]
Wang	2010	46/M	Pubic symphysis	Bladder outlet obstruction	Resection	> 4 years	[5]
Hoshimoto	2000	25/F	Pubic symphysis	Compression and coital pain	Resection	Not mentioned	[6]
Amis	1980	23/M	Pubic symphysis	Asymptomatic	Refused	not mentioned	[7]
Smith	1998	35/M	Pubic ramus	Dyspareunia	Resection	3 months	[8]
Mnif	2009	29/M	Pubic ramus	Dyspareunia	Resection	2 years	[11]
Cardenas	1984	27/M	Pubic symphysis	Asymptomatic	Resection	Not mentioned	[12]
Bacha	2019	35/M	Pubic symphysis	Asymptomatic	Resection	2 years	[13]
Nayak	2018	75/F	Pubic bone	Not mentioned	Not mentioned	Not mentioned	[14]
Lee W	2020	41/F	Pubic symphysis	Pelvic pain, urinary incontinence, urinary frequency, nocturia, dyspareunia	Resection	4 months	[15]
Carpintero^a^	2007	27/M; 37/M	Pubic ramus; ilio-pubic ramus	Dyspareunia; urination difficulty and dyspareunia	Resection; resection	6 years; 2 years	[16]
Peh	1999	43/M	Pubic bone	Groin swelling	Resection	2 years	[17]

^a^This article include two cases, we describe them in sequence

Hematuria caused by osteochondroma of the pubic symphysis represents a rare and unusual clinical presentation. Regarding the present patient, we did not give the correct diagnosis initially due to lack of the awareness of osteochondroma. Of course, the MDT has given us great help in the diagnosis and treatment of this disease. In other words, although osteochondroma is sometimes ignored or misdiagnosed, it can be recognized by careful imaging observation and comprehensive physical examination. Surgical excision is necessary to relieve the associated complications, and the effect of surgery is obvious. We should keep this disease in mind and make a correct diagnosis in similar cases in the future.

## Data Availability

The datasets used and/or analysed during the current study are available from the corresponding author on reasonable request.

## Abbreviations

CT: Computed tomography
HPF: High-power field
MDT: Multi-disciplinary treatment
